# Comparison of the performance of carcinogenic HPV typing of the Roche Linear Array and Qiagen LiquiChip® HPV assays

**DOI:** 10.1186/1471-2334-13-499

**Published:** 2013-10-24

**Authors:** Philippe Halfon, Maria Teresa Sandri, Audrey Raimondo, Sophie Ravet, Hacène Khiri, Mario Sideri, Guillaume Penaranda, Claire Camus, Maria Luisa Mateos Lindemann

**Affiliations:** 1 Laboratoire Alphabio, 1 rue Melchior Guinot, Marseille 13003, France; 2 Hôpital Européen, Marseille, France; 3 Unità Medicina di Laboratorio Laboratory Medicine Unit Milano, Milan, Italy; 4 CDL Pharma, Marseille, France; 5 Servicio de Microbiología, Hospital Ramón y Cajal, Madrid, Spain

## Abstract

**Background:**

Cervical cancer is caused by high-risk types of human papillomavirus (HPV). DNA testing of such high-risk types of HPV could improve cervical screening.The aim of the study was to compare the sensitivities and positive predictive values of two commercially available typing assays (Qiagen LQ and Roche LA) and to comparatively assess the distribution of HPV types with these two assays.

**Methods:**

The study population comprised 311 ASCUS + women with abnormal pap tests who were HCII positive and who were admitted to three European referral gynecology clinics between 2007 and 2010 (Madrid, Marseille and Milan). All patients underwent LQ and LA tests.

**Results:**

The sensitivity of the two assays for HPV typing was 94% for LQ and 99% for LA (compared with HCII). The overall concordance between LQ and LA was 93%. The three prevalent genotypes, HPV16, HPV18, and HPV31, were identified with a high concordance using the two assays: kappa 0.93, 0.83, and 0.91, respectively. Mixed genotypes were more frequently detected by LA than by LQ: 52% vs. 18%, respectively (p < .0001).

**Conclusions:**

These assays have a good clinical sensitivity for detecting HPV types in CIN2+ patients and allow the virus type to be detected in the same experiment. Our study revealed no significant difference between LQ and LA for CIN2+ or CIN3+ diagnosis, indicating similar distributions of HPV types and a mixed genotype detection that is higher for LA than for LQ.

## Background

Cervical cancer is caused by high-risk types of human papillomavirus (HPV). DNA testing of such high-risk (HR) types of HPV could improve cervical screening [1-3]. It has been demonstrated that either HPV HR type 16 or type 18 has a high CIN2+ predictive value [4-6]. The US guidelines suggest that if an FDA-approved genotyping test were available, it would be reasonable to use it in women 30 years old and older with negative cytology results and positive HPV tests. The detection of HPV 16 and/or 18 in that setting would be an indication for referral to colposcopy [7]. In this context, the Linear Array HPV (LA) (Roche) Genotyping Test, which detects 37 HPV genotypes by reverse line blot (RLB) hybridization, correlates in performance to the HPV HCII test for detection of CIN2+ [8-12]. Tools based on different technologies (reverse dot blot: LA, Roche; DNA-chip: Papillocheck® HPV-screening, Greiner; clinical arrays: CLART® Genomica) serve to assess the distribution of the HPV genotypes [13,14]. A novel commercial test (Qiagen LQ®) for the identification of 18 HR-HPV types on GP5+/6 + −PCR products was developed and has been analytically compared to the established RLB genotyping assay [15]. Godinez et al. recently performed clinical validation of LQ in women >40 years old [16]. However, no clinical validation has been performed in women aged >18 years old with an abnormal pap test.The primary aim of our study was to compare the sensitivities and positive predictive values of two commercially available typing assays (LQ and LA) comparatively with HCII using CIN2+ as the clinical cut-off. The secondary aim was to comparatively assess the distribution of HPV types with these two assays.

## Methods

### Patients

The study population comprised 311 ASCUS + women who were admitted to three European referral gynecology clinics between 2007 and 2010: 158 patients from Madrid (Spain), 58 from Marseille (France), and 95 from Milan (Italy). The criteria for eligibility were abnormal cervical smears, HCII-positive result, and referred for colposcopy with histology. A colposcopy, histology, and HCII test were performed independently at each study center (i.e., Madrid, Marseille, and Milan). Before colposcopy, a cervical sample was obtained using the ThinPrep method (Cytyc France Sarl, Roissy, France). The cervical scrapes were collected with the PreservCyt transport medium (Cytyc Corp., Marlborough, MA) and sent to a reference laboratory (Laboratoire Alphabio, Marseille, FRANCE), where the LQ and LA HPV tests were performed. All these tests were performed on the samples collected in PreservCyt liquid media for liquid-based cytology (ThinPrep). Informed consent was obtained from each participant according to ethics committee guidelines. This study was approved by the CPP Sud-Med I (Comité de Protection des Personnes Sud Méditerranée I) under the reference number 07 22.

### Hybrid capture II (HCII) (Digene)

This assay detects 13 HR-HPV genotypes (HPV16, 18, 31, 33, 35, 39, 45, 51, 52, 56, 58, 59, and 68). The HCII test was performed using the automated HCII assay system as previously described (13). Samples with an RLU/CO value ≥1 were considered HCII-HR positive. Samples with an RLU/CO value <1.0 were considered HCII-HR negative.

### Qiagen *HPV genotyping LQ test analysis*

The LQ test utilizes probes for 18 HR-HPV types (i.e., HPV 16,18, 26, 31, 33, 35, 39, 45, 51, 52, 53, 56, 58, 59, 66, 68, 73, and 82) that are linked to color-coded micro-beads [16,17]. The LQ test detection kit procedure was performed in a Luminex 100 IS System (Luminex Corporation), according to the manufacturer’s instructions [18].

### Roche LA

This assay detects 37 HR and low-risk (LR) genotypes individually. They include 15 HR-HPV genotypes (HPV16, 18, 31, 33, 35, 39, 45, 51, 52, 56, 58, 59, 68, 73, and 82), 3 probable HR-HPV genotypes (HPV26, 53, 66), 10 LR-HPV genotypes (HPV6, 11, 40, 42, 54, 61, 70, 72, 81, and CP6108), and 9 genotypes for which the risk is still undetermined (HPV55, 62, 64, 67, 69, 71, 83, 84, and IS39) [9]. The LA test was performed according to the manufacturer’s instructions.

### In-house real-time PCR high-risk HPV genotyping

An in-house Real Time (RT)-PCR genotyping method was performed on LA and LQ discordant cases. All PCR reactions were performed in a 20-μL volume using an ABI PRISM 7000 (Applied Biosystems). Each individual reaction contained 10 μL of Platinum® Quantitative PCR SuperMix-UDG with ROX (Invitrogen, ref 11743), 5 μL of 0.4 μM primers + probes mix (MWG) and up to 5 μL of HPV DNA or sterile DNAse/RNAse-free grade water in non-template controls. The amplification profile was initiated by a 2-minute incubation at 50°C, followed by a 15-minute incubation at 95°C, and a two-step amplification of 15 seconds at 95°C and 60 seconds at 60°C for 45 cycles. For each 96-well PCR microplate, six patients could be tested for the 12 HPV HR (one HPV/well), the HBB gene, and a negative control. To optimize the reproducibility of the test, filling of the plates was conducted using a TECAN Freedom EVO 75 robot. Data were collected during the amplification step using ABI Prism 7000 SDS Software (Applied Biosystem).

In-house real-time PCR detects 12 HR-HPV genotypes: 16, 18, 31, 33, 35, 39, 45, 51, 52, 56, 58, and 59. To validate the performance of our in-house qualitative HPV type detection method using real-time PCR, standard curves were obtained by the amplification of a dilution series of ten million to ten copies obtained from CaSki cell lines for HPV 16, HPV DNA called HPV 475 (sample previously analyzed, infected with HPV18) and DNA extracted from PBMC as a positive control for HBB.

### Statistical analyses

Two-sided P values were calculated by Chi-square or Fisher exact tests and placed on 2 × 2 contingency tables. Kappa statistics were used for the measuring concordance between LQ and LA. The Kappa statistics values range from 0 to 1 (<0.20, poor; 0.21-0.40, weak; 0.41-0.60, moderate; 0.61-0.80, good; and 0.81-1.00, very good) [19]. All P values <0.05 were considered significant. Calculations were performed using SAS software (SAS V9.1, SAS Institute Inc., Cary, NC).

## Results

The key characteristics of the 311 patients are shown in Table 1. The median age was 36 [min 18 – max 79] years; and 142 (46%) patients were at least CIN2. In addition, 129 (41%) patients were CIN1, and 40 (13%) had a normal biopsy. HR-HPV types were detected in 287 (92%) cases with LQ, and in 307 (99%) cases with LA.

**Table 1:** Key characteristics of the study population

**Characteristics**	**ASCUS + patients (N = 311)**
Age; median [min-max]	36 [18–79]
Smear – N (%)	
ASCUS	37 (12)
LSIL	140 (45)
HSIL	133 (43)
Cancer	1 (<1%)
Biopsy – N (%)	
Normal	40 (13)
CIN1	129 (41)
CIN2	83 (27)
CIN3	58 (19)
Cancer	1 (<1%)
Qiagen HPV LQ – N (%)	
Positive (all types)	287 (92)
Negative	24 (8)
Roche LA – N (%)	
Positive (all types)	307 (99)
Negative	4 (1)

Sensitivities, specificities, positive and negative predictive values are reported in Table 2. Both tests displayed good sensitivity (LQ 94% and LA 99%). None of the comparisons of sensitivities and NPVs displayed statistically relevant differences between tests.

**Table 2:** Test performance for CIN2+ histology detection

**Test**	**<CIN2**	**CIN2+**	**Sensitivity (%)**	**Specificity (%)**	**PPV (%)**	**NPV (%)**
Qiagen LQ						
HPV HR Negative	16	8	94	9	46	67
HPV HR Positive	154	133				
Roche LA						
HPV HR Negative	3	1	99	2	46	75
HPV HR Positive	167	140				

Only HPV genotypes common to LQ and LA were considered in the following analysis. Thus, HR-HPV detection was 95% for LQ and 99% for LA. Mixed HPV genotypes were more frequently detected by LA: 52% vs. 18%, respectively, p < .0001.

**Figure 1:**
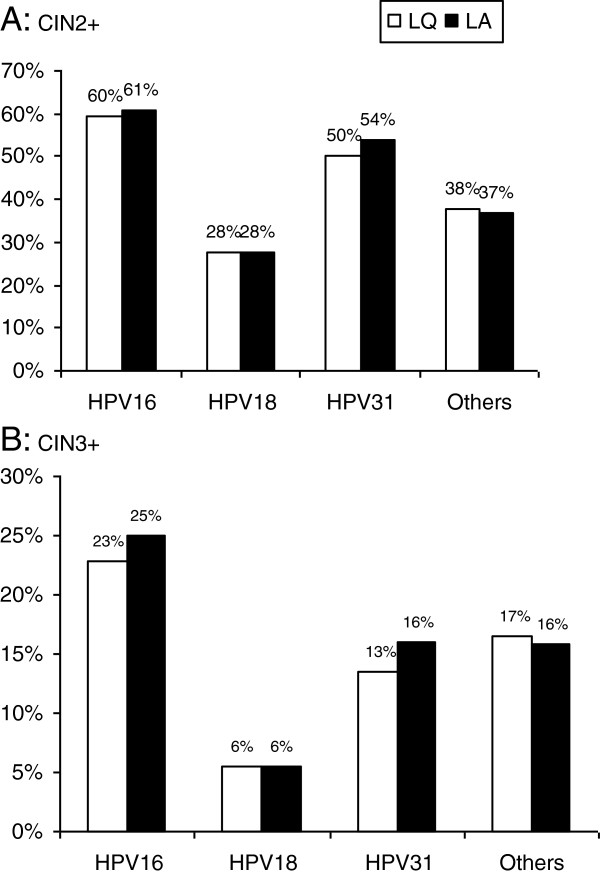
Absolute risks of CIN2+ (A) and CIN3+ (B) according to HPV 16, HPV 18, HPV 31, or other HPV types.

Figures 1A and 1B show the absolute risk of CIN2+ or CIN3+ according to the presence of HPV16, HPV18, HPV31, or other HR-HPV types. HR-HPV types 16, 18, and 31 were chosen because of their high level of oncogenicity and for their high prevalence. The absolute risk of CIN2+ when typed with HPV16 by LQ was 60%, which is similar to the LA risk (61%). When typed with HPV18, the absolute risk of CIN2+ was 28% for both LQ and LA (Figure 1A). Similarly, the absolute risk of CIN3+ when typed HPV16 by LQ was 23%, which is similar to the LA risk (25%). When typed HPV18, the absolute risk of CIN3+ was 6% for both LQ and LA (Figure 1B).

The two tests displayed a high overall concordance rate of 93%. Among the 311 samples, 286 (92%) were HR-HPV positive by both tests, 3 (1%) were HR-HPV negative by both tests, and 22 (7%) were discordant. Among the 21 discordant cases HR-HPV negative by LQ, and seven were CIN2+ based on biopsy. The case that was HR-HPV negative by LA was CIN1 in biopsy. The in-house RT-PCR test was performed on LA and LQ discordant cases (Table 3). Among the 22 discordant cases, 21 were HR-HPV positive by LA, and one was HR-HPV positive by LQ. In-house RT-PCR was in accordance with LA in nine cases and in accordance with LQ in 13 cases.

**Table 3:** Interpretation of the 22 LA/LQ discordant cases using the in house RT-PCR assay

**Sample ID**	**Roche LA HPV genotyping**	**Qiagen LQ**	**In house RT-PCR**	**In house RT-PCR in accordance with:**
DISC1	35	Negative	35	Roche LA
DISC2	35.51.52	Negative	35.51	Roche LA
DISC3	39.54	Negative	39	Roche LA
DISC4	42	Negative	Negative	Qiagen LQ
DISC5	51	Negative	Negative	Qiagen LQ
DISC6	51.81	Negative	51	Roche LA
DISC7	53	Negative	Negative	Qiagen LQ
DISC8	53.59	Negative	Negative	Qiagen LQ
DISC9	54.42	Negative	Negative	Qiagen LQ
DISC10	54.84	Negative	Negative	Qiagen LQ
DISC11	59	Negative	59	Roche LA
DISC12	59	Negative	59	Roche LA
DISC13	59.61	Negative	59	Roche LA
DISC14	59.61.81	Negative	59	Roche LA
DISC15	6.84.42	Negative	Negative	Qiagen LQ
DISC16	61	Negative	Negative	Qiagen LQ
DISC17	67	Negative	Negative	Qiagen LQ
DISC18	67	Negative	Negative	Qiagen LQ
DISC19	67	Negative	Negative	Qiagen LQ
DISC20	68	Negative	Negative	Qiagen LQ
DISC21	81	Negative	56	Roche LA
DISC22	Negative	68	68	Qiagen LQ

Table 4 shows the concordance between LQ and LA according to HPV genotype. The two tests are concordant for the majority of HPV genotypes except for HPV35 (Kappa = 0.41, p = 0.002), HPV53 (Kappa = 0.36, p < .001), HPV59 (Kappa = 0.29, p = 0.02), HPV73 (Kappa = 0.30, p = 0.04), and mixed genotypes (Kappa = −0.08, p < .001). LA detected HPV35 in 11 cases, but the Qiagen assay did not detect HPV35. Among these 11 cases, only two cases were HPV negative by LQ. In the nine remaining cases, LQ detected other HR HPV types. In the same manner, LA detected HPV53 in 35 cases and LQ in eight cases. All eight cases detected HPV53 by LQ were also detected to have HPV53 by LA. LA detected HPV59 in 18 cases. LQ detected HPV59 in three cases, and all were detected by LA. Among the 18 cases with detected HPV59 by LA, five were HPV negative by LQ. HPV73 was the last HPV type for which discrepancies between LA and LQ were significant. LA detected HPV73 in 10 cases and LQ in three cases. Among the three LQ HPV73 cases, one was not detected as HPV73 by LA.

**Table 4:** Concordance between LA and LQ in detecting HR-HPV genotypes

**HPV genotype**	**Roche LA (n)**	**Qiagen LQ (n)**	Kappa* [95% CI]	p-value**
HPV 16	128	131	0.93 [0.89-0.97]	0.60
HPV 18	14	18	0.83 [0.69-0.98]	0.87
HPV 26”	1	1	1.00 [1.00-1.00]	1.00
HPV 31	50	50	0.91 [0.84-0.97]	0.50
HPV 33	26	19	0.83 [0.71-0.95]	0.16
HPV 35	15	4	0.41 [0.13-0.69]	0.002
HPV 39	12	9	0.85 [0.69-1.00]	0.25
HPV 45	14	11	0.88 [0.74-1.00]	0.27
HPV 51	22	14	0.77 [0.61-0.92]	0.15
HPV 52	17	14	0.42 [0.20-0.65]	0.50
HPV 53	35	8	0.35 [0.17-0.52]	<.0001
HPV 56	21	21	0.85 [0.73-0.97]	0.50
HPV 58	27	18	0.79 [0.65-0.92]	0.08
HPV 59	18	3	0.27 [0.03-0.52]	0.02
HPV 66	18	14	0.74 [0.56-0.91]	0.29
HPV 68	8	9	0.58 [0.29-0.86]	0.05
HPV 73	10	3	0.30 [0.00-0.62]	0.04
HPV 82‴	1	1	1.00 [1.00-1.00]	1.00
Mixed	163	56	−0.08 [−0.15-0.02]	<.0001

*: Kappa statistic (<.20, poor; 0.21-0.40, weak; 0.41-0.60, moderate; 0.61-0.80, good; 0.81-1.00, very good). ** Measures significance of the difference between LQ and LA. ″: Sample positive for HPV 26 was the same for LQ and LA. ‴: Sample positive for HPV 82 was the same for LQ and LA.

## Discussion

In this study, an LQ HPV genotyping test was compared to the widely used LA test. LQ and LA were similar in HR-HPV detection (92% of concordant HR-HPV positive results). Although all samples were HCII positive, HPV negative results using the LA assay were found in 4 (1%) cases, compared with 24 (8%) for the LQ assay. Among the 311 samples, 286 (92%) were HR-HPV positive by both assays, 3 (1%) were HR-HPV negative by both assays, and 22 (7%) were discordant. Among the 22 LA and LQ discordant cases, in-house RT-PCR was in accordance with LA in nine cases and with LQ in 13 cases. A reason for the discordance between LA and LQ might be that among the 21 HPV-positive cases detected by LA but not by LQ, there were nine cases in which LA detected HR-HPV types LQ was not designed to detect.

The LA and LQ tests were concordant in detecting the majority of HR-HPV genotypes except HPV35, HPV53, HPV59, and HPV73. The three prevalent genotypes, HPV16, HPV18, and HPV31, were found with a high concordance between LA and LQ: kappa 0.93, 0.83, and 0.91, respectively. These findings corroborate those reported by Godinez et al. [16]. All the cases where the listed HPV types were detected by LQ (35, 53, 59, and 73) were also detected by LA, except for one case where LQ detected HPV73 but not LA. Godinez et al. reported overestimation of LA for HPV52 and HPV53 because of cross-reactivity with other probes. We think it might explain the discrepancies between LA and LQ in detecting HPV35, HPV53, HPV59, and HPV73 [16]. Mixed genotypes were more frequently detected by LA than LQ: 52% vs. 18%, respectively (p < .0001). As previously reported, LA is known to have a higher power in identifying mixed genotypes, but the clinical implication of this higher power is not well established [8,9,16]. One hypothesis could be that the genotyping results are due to the nature of the genotype detection used by the test: LA uses a strip that is known to be more sensitive to detect a small quantity of DNA, instead of the liquid used with the LQ.

The analysis of absolute risk of CIN2+ and CIN3 revealed no difference between LA and LQ. The two assays are very similar, regardless of HPV type. As an example, the absolute risk of CIN2+ when HPV16 positive (60%) is approximately two-fold the absolute risk of CIN2+ when HPV18 positive (28%).

The overall agreements between LA and LQ genotyping assays indicated highly similar outcomes and were close to those in recent reports using different genotyping methods [8,9,20-23]. However, the HR-HPV positivity rate was higher (not statistically) with the LA assay than with the LQ.

Genotype-specific results obtained with LA and LQ were highly similar, with 91% of concordant or compatible results.

Regarding the laboratory technical characteristics, LQ® uses multiplex, bead-based xMAP technology and an automated, high-throughput read-out with either the LiquiChip 200 workstation (QIAGEN, Hilden, Germany) or Luminex 100 IS System (Luminex Corporation, Austin, TX). The test was developed for identification of the 18 HR-HPV genotypes associated with cervical cancer by using GP5+/6 + −PCR products. A high level of agreement has been reported between the LQ test and the LA assay for detection and genotyping of 18 HR-HPV types in HCII positive specimens [15].

A limitation of this study might be its relative low number of patients included despite the specific inclusion criteria.

## Conclusions

In conclusion, LQ and LA assays both have good clinical sensitivity for detecting HPV types in CIN2+ adult women. This study revealed no significant difference between LQ and LA for CIN2+ or CIN3+ diagnosis, in addition to indicating similar distributions of HPV types. These assays detect and determine the type of virus in the same experiment. The availability of HPV genotyping methods with demonstrated reliable clinical performance is a necessary prerequisite for HPV genotyping assays.

## Competing interests

The authors declare that they have no competing interests.

## Authors’ contributions

PH conceived the study, participated in its design and coordination, recruited patients, and drafted the manuscript. MTS, MS, and MLML participated in the design of the study and coordination, recruited patients, and helped to draft the manuscript. AR, SR, HK, and CC performed the sample analyses. GP performed the statistical analyses and drafted the manuscript. All authors read and approved the final manuscript.

## Pre-publication history

The pre-publication history for this paper can be accessed here:

http://www.biomedcentral.com/1471-2334/13/499/prepub
